# DNA Sliding Clamps as Therapeutic Targets

**DOI:** 10.3389/fmolb.2018.00087

**Published:** 2018-10-22

**Authors:** Amanda S. Altieri, Zvi Kelman

**Affiliations:** ^1^Institute for Bioscience and Biotechnology Research, University of Maryland and the National Institute of Standards and Technology, Rockville, MD, United States; ^2^Biomolecular Labeling Laboratory, Institute for Bioscience and Biotechnology Research, National Institute of Standards and Technology, Rockville, MD, United States

**Keywords:** β-clamp, DNA clamp, DNA sliding clamp, proliferating cell nuclear antigen, PCNA, therapeutic

## Abstract

Chromosomal DNA replication is achieved by an assembly of multi-protein complexes at the replication fork. DNA sliding clamps play an important role in this assembly and are essential for cell viability. Inhibitors of bacterial (β-clamp) and eukaryal DNA clamps, proliferating cell nuclear antigen (PCNA), have been explored for use as antibacterial and anti-cancer drugs, respectively. Inhibitors for bacterial β-clamps include modified peptides, small molecule inhibitors, natural products, and modified non-steroidal anti-inflammatory drugs. Targeting eukaryotic PCNA sliding clamp in its role in replication can be complicated by undesired effects on healthy cells. Some success has been seen in the design of peptide inhibitors, however, other research has focused on targeting PCNA molecules that are modified in diseased states. These inhibitors that are targeted to PCNA involved in DNA repair can sensitize cancer cells to existing anti-cancer therapeutics, and a DNA aptamer has also been shown to inhibit PCNA. In this review, studies in the use of both bacterial and eukaryotic sliding clamps as therapeutic targets are summarized.

## Introduction

DNA polymerases that replicate chromosomal DNA are not processive by themselves and polymerize only a few nucleotides at a time. In all organisms, processive replication is achieved by additional factors including a protein referred to as a “sliding clamp”. The sliding clamp is a ring-shaped protein that encircles duplex DNA, binds to the DNA polymerase and tethers it to the DNA template, preventing its dissociation and providing high processivity. The sliding clamp does not assemble itself around DNA, but is loaded onto DNA in an ATP-dependent mechanism by a “clamp loader” complex. In all organisms, the sliding clamps and clamp loaders are essential for cell viability. In addition to their role in chromosomal DNA replication, the sliding clamps also play essential roles in DNA repair, recombination, and cell cycle progression and control (Kelman and O'Donnell, 1995; Jeruzalmi et al., 2002; Vivona and Kelman, 2003). In both bacteria and eukarya, many proteins interact with sliding clamps and these interactions regulate their biochemical properties (Kelman and Hurwitz, 1998; Vivona and Kelman, 2003).

Proliferating cell nuclear antigen (PCNA) is the eukaryotic sliding clamp (Kelman, 1997; Moldovan et al., 2007) and plays an essential role in chromosomal DNA replication, repair and recombination as well as other cellular processes such as translesion synthesis (Yang and Gao, 2018). PCNA forms a stable homotrimer. Replication factor C (RFC) utilizes the energy from ATP hydrolysis to assemble trimeric PCNA around duplex DNA at the primer-template junction on the lagging strand. PCNA interacts with the two replicative polymerases, DNA polymerases δ and ε (Polδ and Polε), proteins involved in Okazaki fragment maturation [i.e., DNA ligase and flap endonuclease-1 (FEN-1)], proteins needed for DNA repair [Apurinic/apyrimidinic endonuclease 1 and Xeroderma pigmentosum G, cell cycle regulators (i.e., p21)] and many other cellular factors. The DNA repair PCNA proteins are also often post-translationally modified, and the type of post-translational modification directs PCNA towards different signaling processes (Wang, 2014).

DNA polymerase III (Pol III) is the replicative polymerase in bacteria. The β-subunit of Pol III is the bacterial sliding clamp, also called the β-clamp (Kuriyan and O'Donnell, 1993; Kelman and O'Donnell, 1995). It forms a stable dimer and requires the τ-complex for assembly around the primer-template junction in an ATP-dependent manner. Similar to eukaryal PCNA, the β-clamp interacts with the replicative polymerase, Pol III, as well as proteins involved in DNA repair (i.e., Pol II and Pol IV), the cell cycle regulator, DnaA, and other proteins.

**Figure 1:**
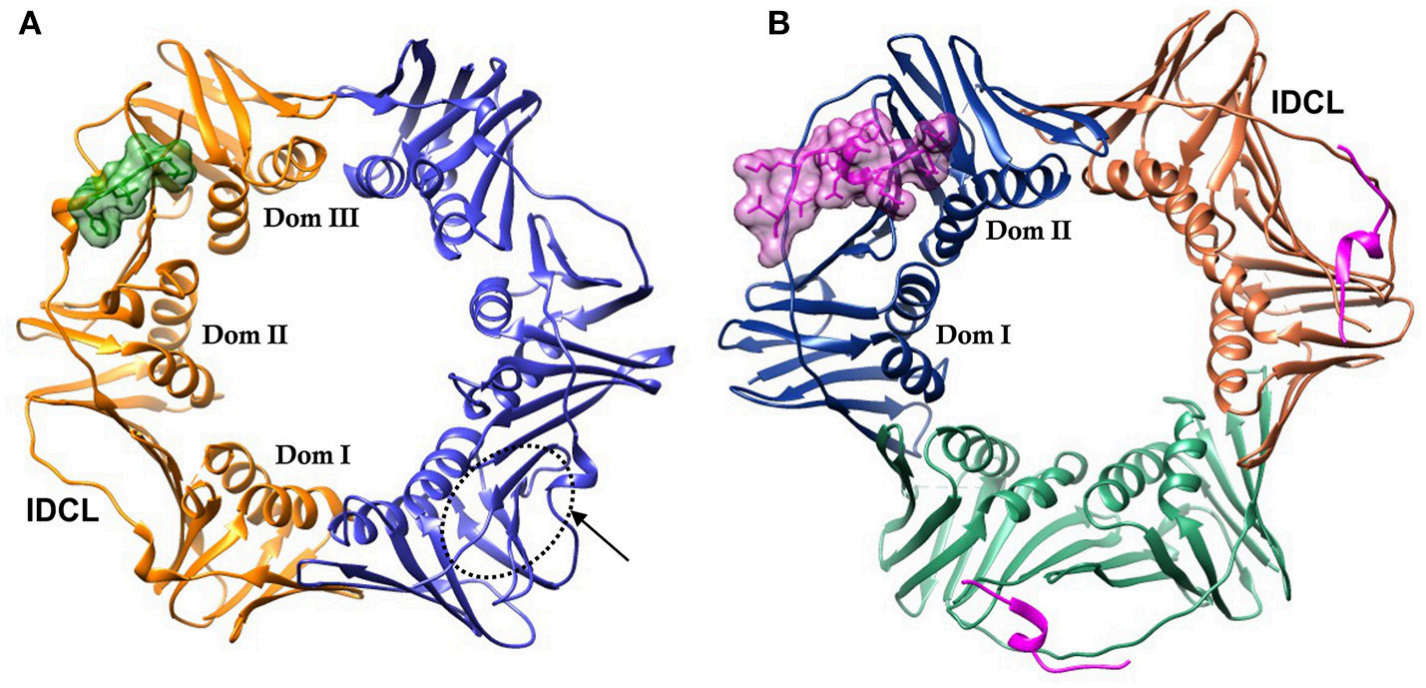
DNA sliding clamps showing pseudo-hexameric symmetry and the central hole of the ring structure that accommodates double stranded DNA. **(A)** The *Escherichia coli* β-clamp dimer with one monomer colored blue and the other monomer orange. The three similar domains in each monomer are labeled Dom I, II, and III. One of the four IDCL loops is labeled. The peptide AcQADLF with its surface colored green shows the location of one of the binding pockets. The second binding pocket, notated by an arrow and dotted line is empty in this structure [PDBID: 4K30 (Zhao et al., 2013)]. **(B)** The human PCNA trimer with one monomer in orange, one in green and the third monomer colored blue. The two domains in one of the monomers are labeled Dom I and II. One of the three IDCLs is labeled. The FEN-1 PIP peptides are drawn in purple, with one of the ligands shown in molecular surface representation and the other two ligands as ribbons [PDBID: 1U7B (Bruning and Shamoo, 2004)]. Molecular rendering was made using Chimera (Pettersen et al., 2004).

DNA sliding clamps from all organisms share a common architecture. They are multi-domain, multimeric proteins that form a toroidal structure with an ~35 Å diameter central pore large enough to accommodate duplex DNA that is lined with positively charged side chains, primarily Lys and Arg. PCNA is a trimeric protein, while the β-clamp is dimeric. Although there is low sequence identity between PCNA and β-clamp (< 15%), their three-dimensional structures are nearly superimposable. This comes about from the similar structure of the domains that comprise each chain. These domains consist of two 4-stranded β-sheets that are located on the outside of the DNA clamp, and two α-helices that when assembled, line the core of the clamp. There are three domains in each monomer of the β-clamp dimer, and two domains in each monomer of the PCNA trimer, creating a pseudo-hexameric symmetry that is present in all DNA clamps (Kelman and O'Donnell, 1995) (Figure 1).

The face of the DNA clamp that points in the direction of DNA synthesis is known as the front face, and is the interaction site for many binding partners. The interaction sites on DNA clamps are largely hydrophobic and are located near domains I and II in PCNA and domains II and III in β-clamp (Figure 1). Most proteins that interact with PCNA do so via a conserved motif referred to as the PCNA interacting peptide (PIP) motif (Warbrick, 1998; Warbrick et al., 1998). These PIP motifs are short protein segments that are usually located at the C-terminal end of the interacting proteins. For example, the p21 protein functions as an inhibitor of the cyclin-dependent protein kinases that control the initiation of the cell cycle S phase and DNA replication. PCNA interactions with p21 or the C-terminal peptide of p21 can inhibit other proteins binding to PCNA and affect PCNA activities (Gibbs et al., 1997). The C-terminal peptide of p21 contains a PIP motif and binds to the PIP site (Gulbis et al., 1996). The PIP motif is a weak consensus of Qxxhxxaa, where “h” is a hydrophobic amino acid (isoleucine, leucine or methionine) and “a” is an aromatic residue (tryptophan, tyrosine, or phenylalanine). Although the PIP motif is the most common interaction sequence among PCNA-interacting proteins, other motifs have also been reported to bind PCNA. For example, the AlkB homolog 2 PCNA-interacting motif (APIM) is commonly found in DNA repair enzymes (Gilljam et al., 2009). The APIM is a five residue motif, (K/R)(F/Y/W)(L/I/V/A)(L/I/V/A)(K/R), which binds at the PIP site in a similar conformation to PIP peptides (Sebesta et al., 2017). Much of the research on therapeutics to PCNA is focused on its role in DNA repair often in combination with other therapeutics (Gederaas et al., 2014; Inoue et al., 2014).

The analogous binding sequence to bacterial DNA clamps is a five residue linear motif with a canonical sequence of QL(S/D)LF (Dalrymple et al., 2001) and is called β-clamp binding motif (CBM). The peptide sequences that bind to the bacterial and eukaryal DNA clamps share a few similarities, namely an N-terminal Q and two hydrophobic, often aromatic amino acids at the peptide C-terminus. However, the total number of amino acids in the clamp binding sequences is different (eight for PCNA and five for the β-clamp) and there is no similarity between the remaining residues. It is no surprise, then, that the peptide pockets at each of the DNA clamps are significantly different. As such, peptides that bind to the β-clamp do not bind to PCNA and vice versa (Flores-Rozas et al., 1994).

Since DNA clamps operate as a binding “hub” with many interacting proteins (Kelman and Hurwitz, 1998), they show a certain amount of binding site promiscuity. Specificity to the binding pocket depends on conserved residues of the peptide motif binding to target receptor “hot spots” (Yin et al., 2013). Any effective DNA clamp inhibitor must bind tightly in order to inhibit DNA synthesis (Wolff et al., 2011) or repair. Initial leads for compounds that bind to the peptide pockets on sliding clamps are often discovered using high-throughput screening of compound libraries to identify molecules that bind to the interaction site. Further modification of these lead compounds to optimize binding to the pocket is measured using cell assays, affinity measurements and structural details. DNA sliding clamps are essential for cellular replication and repair, and as such, are a prime target for the development of anti-proliferatives and antibacterial drugs. The current world-wide emergence of antibacterial resistance and prevalence of cancer make these efforts current and crucial. In this review, studies on DNA sliding clamps as drug targets are summarized. Previous reviews related to the subject include (Bruck and O'Donnell, 2001; Kontopidis et al., 2005; Wang, 2014; Choe and Moldovan, 2016).

## The β-clamp

**Figure 2:**
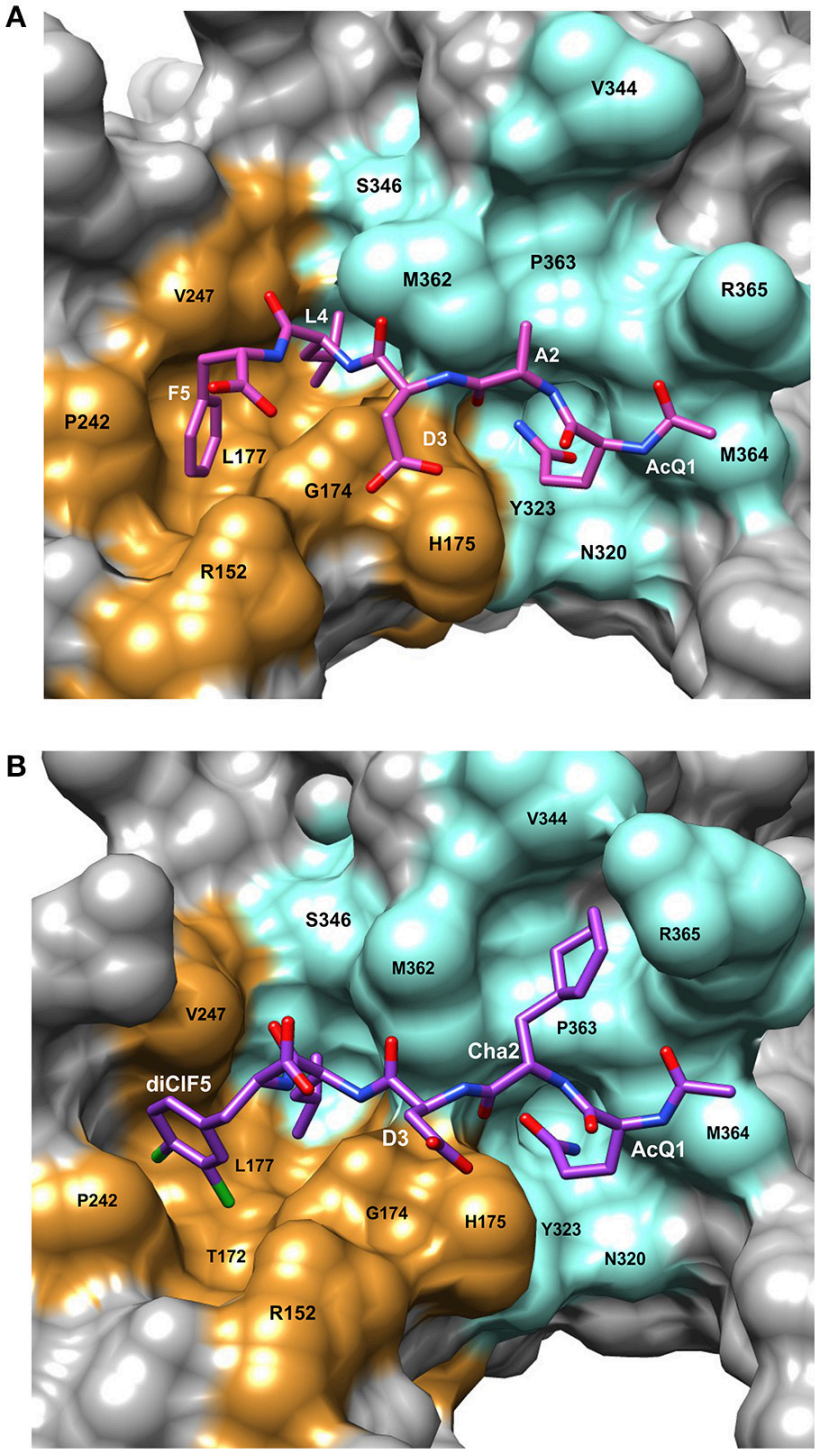
Details of the peptide binding site on β-clamp. The surface residues in subsite 1 are colored in orange and subsite 2 in cyan. Residue labels in white are for the CBM peptide and labels in black are the β-clamp binding site residues. **(A)** Surface representation of the peptide binding pocket and stick figure of the AcQADLF peptide bound to *E. coli* β-clamp [PDBID: 4K3O (Zhao et al., 2013)]. **(B)** Surface representation of the *E. coli* β-clamp peptide binding pocket with a modified peptide competitive inhibitor, Ac-Q_1_Cha_2_D_3_L_4_(3,4)ClF_5_ bound in the peptide pocket [PDBID: 3Q4L (Wolff et al., 2011)]. Molecular rendering was made using Chimera (Pettersen et al., 2004).

The bacterial β-clamp is a homo-dimer of ~82 kDa. As described above, each monomer of β-clamp is composed of three similar globular domains resulting in an overall pseudo-hexameric symmetry (Kong et al., 1992). The domains of each monomer have extensive interactions along the neighboring β-strands and α-helices. In addition, there are four flexible inter-domain connector loops (IDCLs) between the domains, two for each monomer. The dimer interface consists of β-strand contacts in a head to tail arrangement (Figure 1A). The peptide binding pocket for CBMs is located near the IDCL of domains II and III, and consists of two subsites: subsite 1 between domains II and III that is ~8.5 Å deep, and subsite 2 in domain III that is narrower and shallower at ~4.5 Å deep. Using the numbering 1 through 5 to refer to the five canonical residues of the CBM (Q_1_L_2_(S/D)_3_L_4_F_5_), peptide ligand residues L_4_ and F_5_ bind to subsite 1, while residues Q_1_ and L_2_ bind to subsite 2 (Bunting et al., 2003; Burnouf et al., 2004; Georgescu et al., 2008). Most of the interactions between the peptide and the binding pocket are hydrophobic. However, there are several β-clamp side chains that make ionic contacts to the peptide ligand as well as a few backbone amides and carboxyl oxygens that form hydrogen bonds to the peptide. An example of a linear peptide in the binding pocket is shown in Figure 2A [PDBID: 4K30 (Zhao et al., 2013)]. This structure contains the peptide sequence Ac-QADLF, but it is clear that the binding pocket can also accommodate the more canonical L_2_ in the hydrophobic pocket in subsite 2. Many bacterial proteins bind to β-clamp at the CBD through similar interactions.

## Peptides as inhibitors of β-clamp

Peptides from the C-terminus of the Pol III δ subunit that bind to the β-clamp compete effectively with binding of the intact δ subunit (Yin et al., 2013). Peptides are synthesized using known chemistry and as drugs can have low toxicity and good efficacy, however they are often quickly metabolized. It was shown that this problem can be overcome by conjugating a fatty acid to the amino acid sidechain of a short peptide. The fatty acid is a ligand to stable blood-serum proteins such as albumin, and when the fatty acid binds to albumin it helps protect the peptide-drug in circulation in the body. These new, soluble “chimera ligands” bind tightly to human albumin (K_D_ ~ 40 nM) and can be attached to peptide drugs using standard synthesis (Zorzi et al., 2017).

Several β-clamp inhibitors were designed that are modified CBM peptides. The goal was to maintain the structure of the canonical peptide backbone in the binding pocket, while increasing the affinity to β-clamp by varying the side chains with other moieties and non-natural amino acids. A commonly used modification is the acetylation of Q_1_ (to form Ac-Q_1_L_2_D_3_L_4_F_5_) that improves binding by about 30-fold compared to the non-acetylated peptide, due to hydrogen bond formation to an arginine residue in subsite 2 (Yin et al., 2013). When F_5_ is replaced with a 3,4-dichlorophenyalanine, the resulting Ac-Q_1_L_2_D_3_L_4_(3,4)ClF_5_ peptide binds with three times higher affinity (Table 1), due to improved hydrophobic and van der Waal's contacts from the halogen groups to subsite 1. The combination of the Ac-Q_1_ and (3,4)ClF_5_ substitutions results in 110-fold tighter binding for this modified penta-peptide inhibitor (Wijffels et al., 2011).

**Table 1:** Inhibitory constants of β-clamp ligands^a^^,^^b^^,^^c^.

**Ligand**	**Molecule**	**IC_50_ (μM)**	**K_i, d_ (μM)**	**Method**	**References**
**NATURAL PEPTIDE**					
Pol IV peptide (P1)	R_0_Q_1_L_2_V_3_L_4_G_5_L_6_	8.85	0.15	SPR to β-clamp	Wolff et al., 2011
Consensus	Q_1_L_2_D_3_L_4_F_5_	63.2	29.5	FP to β-clamp	Yin et al., 2013
**MODIFIED PEPTIDES**					
Ac-consensus	Ac-Q_1_L_2_D_3_L_4_F_5_	1.9	0.9	FP to Pol III β-clamp	Yin et al., 2013
Ac-consensus	Ac-Q_1_L_2_D_3_L_4_F_5_	0.07	n.r.	α-subunit plate binding	Wijffels et al., 2011
Ac-consensus (P6)	Ac-Q_1_L_2_D_3_L_4_F_5_	1.12	1.2	SPR to β-clamp	Wolff et al., 2011
Ac-cons.+diClF_5_	Ac-Q_1_L_2_D_3_L_4_(3,4)ClF_5_	0.021	n.r.	α-subunit plate binding	Wijffels et al., 2011
P14	Ac-Q_1_Cha_2_D_3_L_4_(3,4)ClF_5_	0.077	i, 17	SPR to β-clamp	Wolff et al., 2011
**SMALL MOLECULES**					
RU-7	RU-7	n.r.	i, 10	Pol III replication assay	Georgescu et al., 2008
O-8	compound 8	115	i, 64	screen, x-ray structure	Yin et al., 2014a
Compound 4	compound 4	40	n.r.	α-subunit plate binding	Wijffels et al., 2011
**NATURAL PRODUCT**					
CGM	Pro-8-cylohexanyl GM	n.r.	0.66	SPR	Kling et al., 2015

a *FP, fluorescence polarization; SPR, surface plasmon resonance*.

b *n.r., value not reported*.

c *Dissociation constants are reported in the original literature sometimes as Ki and in others as Kd. Kd's are listed except where noted as Ki*.

Another example of β-clamp inhibitor design based on the native peptide started with the polymerase IV CBM peptide, R_0_Q_1_L_2_V_3_L_4_G_5_L_6_ (called P1). The first peptide modifications were acetylation of Q_1_ (described above) and use of the consensus peptide (P6) which improved affinity to β-clamp. Modification of L_2_ to a cyclohexyl-L-alanyl (Cha) residue and F_5_ to 3,4-dichlorophenylalanine improved binding by an additional 15-fold (Wolff et al., 2011). Overall, this modified peptide inhibitor, Ac-Q_1_Cha_2_D_3_L_4_(3,4)ClF_5_ (called P14), bound 100 times tighter than the native P1 (Table 1). The structure of P14 bound to the β-clamp (Figure 2B) shows the backbone is in a similar conformation as the native peptide (Wolff et al., 2011). The tighter affinity is achieved because the Cha residue extends further into the hydrophobic pocket and makes additional subsite 1 interactions. In addition to stronger hydrophobic contacts from the halogen-substituted phenylalanine, the meta-chlorine forms a halogen bond with the hydroxyl oxygen of a threonine providing further enhanced binding (Wolff et al., 2011).

## Small molecules as inhibitors of β-clamp

One of the first β-clamp inhibitors reported was identified using library screening for compounds that inhibited *in vitro* DNA synthesis by Pol III and competed for binding to β-clamp. This compound, called RU7, contains a di-brominated aromatic ring and also had different inhibitory effects on Pol II, Pol III, and Pol IV. The structure of the RU7-β-clamp complex showed that RU7 was bound to subsite 1 in the binding site, but had fewer overall contacts to the pocket than the native CBM peptide (Georgescu et al., 2008). Another small molecule inhibitor that was identified from an *in silico* screen of the D_3_L_4_F_5_ tripeptide motif, is a biphenyloxime ether peptide mimetic, called “compound 4” (Table 1) (Wijffels et al., 2011). Another set of inhibitors was designed using structurally based fragment screening and other *in silico* methods. A resulting lead, called “compound 8,” is a tetrahydrocarbazole and inhibited both gram-negative and gram-positive bacteria (Table 1). Improved efficacy was achieved by increasing the number of contacts within subsite 1 of the β-clamp (Yin et al., 2014a).

## A natural product as an inhibitor of β-clamp

Griselimycin (GM) is a bacterial derived natural product that was isolated from *Streptomyces* and has antibacterial activity against *Mycobacteria*. GM is a partially cyclic peptide with the sequence V_1_P_2_T_3_L_4_P_5_L_6_V_7_P_8_L_9_G_10_ where the cyclization is between T3 and G10. Initially it was not fully developed into a drug product because of a short half-life upon oral administration, but it was revisited in order to address drug-resistant strains of *Mycobacterium tuberculosis* (Kling et al., 2015). Metabolic stability profiling experiments of natural analogs showed that the eighth residue in GM, a proline, is the site of metabolic degradation and the cause of instability. Two modifications of Pro_8_ at the Cδ atom on GM caused it to be more resistant to degradation without affecting the binding affinity: one is addition of a methyl group resulting in methyGM and the other is a cyclohexanyl group, forming cyclohexylGM (CGM). A crystal structure of CGM bound to β-clamp of *M. tuberculosis* showed that it binds in the CBM peptide interaction site, inhibits the interaction of β-clamp with the Pol III δ subunit and may also lead to reduced polymerase processivity. Most of the interactions between CGM and the β-clamp are hydrophobic, with only two hydrogen bonds. Interestingly, low frequency resistance to GM was seen in *M. tuberculosis* and *Mycobacterium smegmatis* that was attributed to up-regulation of several genes. One of these genes is the gene encoding for β-clamp, the *dnaN* gene. It was found that the overexpression of the *dnaN* gene in low-frequency resistance is due to a point mutation in the *dnaN* promotor resulting in an elevated level of β-clamp (Kling et al., 2015). This suggests that Mycobacterial resistance to GM is mediated by over expression of β-clamp.

## Non-steroidal anti-inflammatory drugs (NSAIDs) as inhibitors of β-clamp

Several non-steroidal anti-inflammatory drugs exhibited suppression of *Escherichia coli* Pol III β-clamp (Yin et al., 2014b). An assay using minimal components (β-clamp, the clamp-loader complex, Pol III α subunit and single-stranded binding protein), showed that inhibition of β-clamp-mediated interactions by these NSAIDs directly affected *E. coli* DNA replication *in vitro*. Crystal structures of three NSAID-β-clamp complexes showed that carprofen, bromfenac, and vedaprofen bound to subsite 1 on β-clamp. These molecules bury a hydrophobic group into subsite 1 and an aromatic group into an adjacent region. The lack of interaction with subsite 2 is likely the reason for the relatively weak interaction with β-clamp (minimal inhibitory concentration, MIC > 1,250 μM) and relatively weak inhibition as compared to antibiotics such as ampicillin (MIC = 125 μM) and Chloramphenicol (MIC = 1.37 μM). Nevertheless, these results suggest that NSAID drugs may be used as a promising starting point for the design of new antibiotic drugs (Yin et al., 2014b).

## Considerations for therapeutics targeted to β-clamp

Different ligands that bind at the protein-protein interaction site on β-clamp were discussed above. Although a consensus sequence has been identified, the CBM of various bacterial proteins have somewhat different sequences and number of residues [for example see: (Patoli et al., 2013)]. The overall structure of the β-clamp does not change in many of these binding events, as the root-mean-square deviation (r.m.s.d.) between bound and ligand free structures is between 1 and 3 Å. There are local changes, however, in the β-clamp binding pocket to accommodate the ligand. In the *E. coli* β-clamp, there is a rotation of the M_362_ and S_346_ sidechains that opens a pathway between subsites 1 and 2 upon ligand binding. In addition, the β-clamp sidechain R_365_ shifts position and opens a hydrophobic platform for the canonical L_3_ residue (Wijffels et al., 2011). Any effective inhibitors that are designed to this pocket should interact with both subsites.

In addition to the design of inhibitors that have higher affinity than the natural ligand, specificity of the drug to its molecular target and also to the bacterial species is important to determine the activity spectrum of the drug. For example, differences were found between GM analogs and their interaction with different strains of *Mycobacteria*. GM also has lower binding to *E. coli* β-clamp and does not interact with eukaryotic PCNAs (Kling et al., 2015). These results imply that it may be possible to target drugs to an individual bacterial strain. A study assessing the different modes of peptide binding to the β-clamps from various bacteria found that there are differences in the binding thermodynamics of the peptides to their cognate clamps, and that small modifications can greatly affect the affinities (Wolff et al., 2014). Development of a new, successful antibacterial drug will likely require a combination of multiple approaches, including those discussed above.

## Proliferating cell nuclear antigen (PCNA)

**Figure 3:**
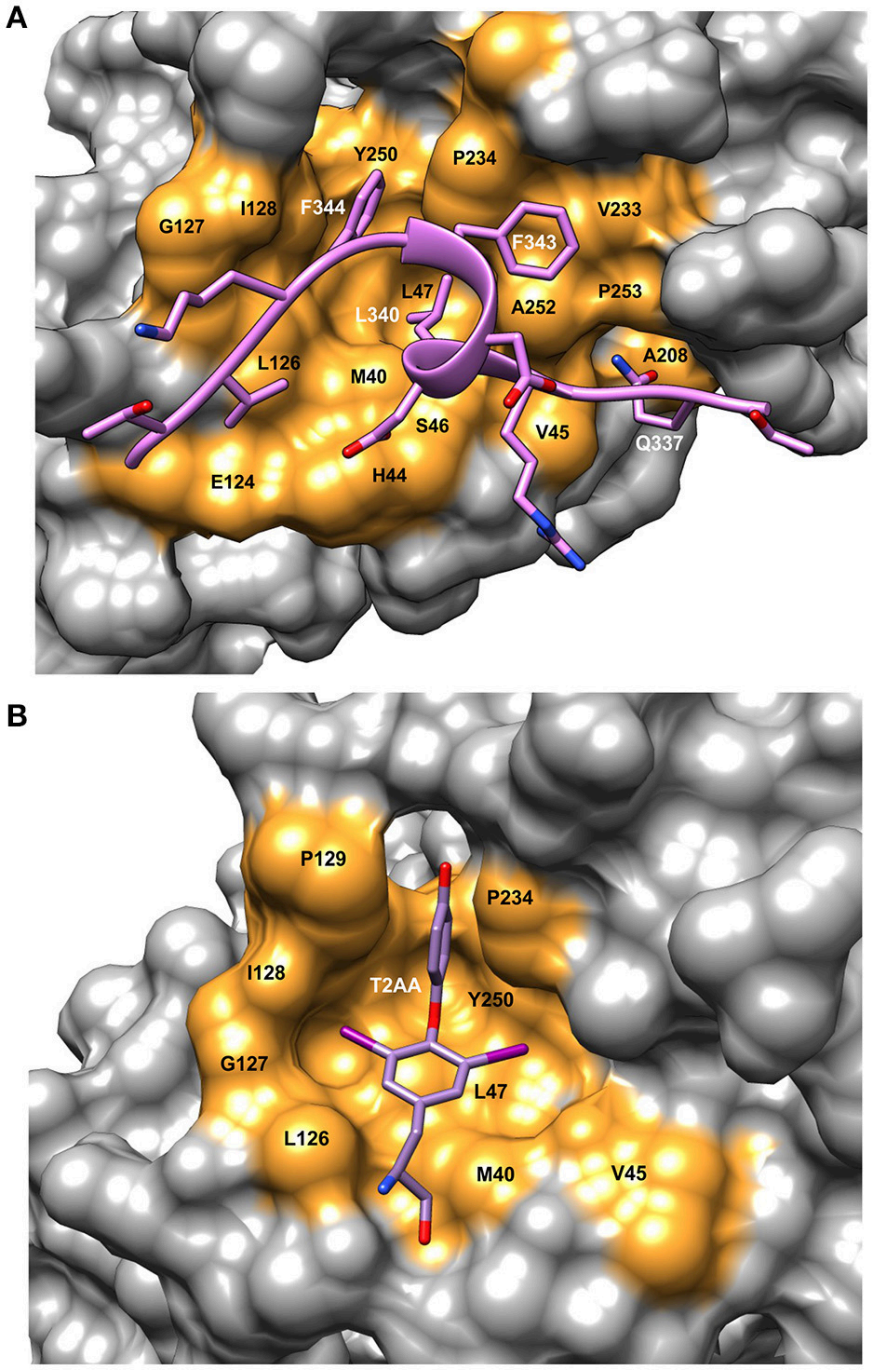
Details of the binding site on PCNA showing ligand interactions. **(A)** Surface representation of FEN-1 peptide bound to human PCNA [PDBID:1U7B (Bruning and Shamoo, 2004)]. Residue labels in white refer to the FEN-1 peptide and labels in black indicate PCNA binding site residues. **(B)** Surface representation of the PIP binding pocket of mono-ubiquitinated human PCNA with the small molecule inhibitor, T2AA bound [PDBID: 3WGW (Inoue et al., 2014)]. This inhibitor binds 2:1 to PCNA with the second binding site at the interface between the subunits (detail not shown). Molecular rendering was made using Chimera (Pettersen et al., 2004).

PCNA is a homo-trimeric protein of ~86 kDa. The two domains in each monomer are connected with a flexible loop referred to as the IDCL as in the β-clamp. The PIP sequence interacts with a hydrophobic pocket on the front face of the PCNA protein near the IDCL (Gulbis et al., 1996) (Figure 1B). The PIP peptide binding pocket on PCNA consists of a “Q” pocket in which hydrogen bonds form between the conserved Q sidechain and PCNA residues, which is then followed by a hydrophobic patch (Figure 3A). The PIP peptides generally form a single turn of a 3–10 helix that begins at the hydrophobic fourth residue and places this residue next to the final two hydrophobic, aromatic residues (7 and 8). These three residues fit into the hydrophobic pocket in an orientation resembling a plug, as shown for a PCNA-interacting protein FEN-1 (Bruning and Shamoo, 2004) (Figure 3A). Most of these interactions are conserved among PIP peptides and proteins that bind to PCNA.

PCNA was originally identified as a nuclear antigen in highly proliferating cells hence the name proliferation cell nuclear antigen (Miyachi et al., 1978; Bravo and Celis, 1980). Therefore, PCNA is a target for the development of anti-proliferation and anti-cancer drugs. In cancer treatment, chemotherapy can be genotoxic to normal cells as well as cancer cells. By specifically targeting proliferative cancer cells, the toxicity of these treatments is decreased. In its role in translesion synthesis, PCNA is post-translationally modified (Wang, 2014) and is not associated with chromatin. It is possible that developing therapeutics to these modified PCNA molecules will lead to more specific targeting of diseased cells. Many PCNA inhibitors bind at the PIP-site and prevent other protein partners from binding thus inhibiting DNA replication. However, not all PCNA inhibitors bind at the PIP pocket. One of the small molecule inhibitors, called PCNA-I1, was shown to bind instead to the interface between PCNA monomers resulting in reduced PCNA binding to chromatin (Tan et al., 2012).

## Peptides as inhibitors of PCNA

PCNA function and its interaction with APIM are important during cellular stress as they play a role in the repair of damaged DNA. Therefore, inhibiting this interaction can affect the survival of cells undergoing stress induced by chemotherapeutic drugs (Gederaas et al., 2014). Over-expression of APIM-containing peptides caused cancer cells to be hypersensitive against various chemotherapeutics. ATX-101 is a cell-penetrating APIM-containing peptide (Muller et al., 2013) and was shown to increase the anticancer efficacy of the drug mitomycin C that creates inter-strand crosslinks in DNA, as well as with bleomycin and gemcitabine in bladder cancer cells (Gederaas et al., 2014). Similarly, ATX-101 induced rapid caspase-dependent apoptosis and increased the cytotoxic effect of melphalan over several days in multiple myeloma cells. Treatment with ATX-101 induced apoptosis in all phases of the cell cycle. This is different from the activity of two small molecule PCNA inhibitors of cancer cell growth that disrupt PCNA during replication, T2AA and PCNA-I1 (Punchihewa et al., 2012; Tan et al., 2012), and rely on the high proliferation rate of these cells (Muller et al., 2013; Choe and Moldovan, 2016).

Another modified PCNA binding peptide has shown promise in breast cancer. Analysis of breast cancer tissue showed increased PCNA expression over normal tissue nearby and this type of cancer was also correlated with shorter survival (Smith et al., 2015). DNA replication in malignant breast cancer cell lines and tumor tissue is more error-prone than in non-malignant tissue (Sekowski et al., 1998). A unique form of PCNA, termed cancer-associated PCNA (caPCNA) was identified in these cells that is different from PCNA in normal breast cells due to a post-translational modification, specifically methyl esterification of glutamic and aspartic acids residues (Hoelz et al., 2006). Using a polyclonal antibody that specifically recognizes the caPCNA isoform, a peptide sequence was identified, called R9-cc-caPeptide (cc is a linker from R9 to the caPeptide), and was synthesized to mimic this region. The nine arginines (R9) were added to facilitate uptake across the cell membrane. The designed peptide part of the sequence, caPeptide, corresponds to residues 126–133 of PCNA, which is part of the IDCL. The caPeptide inhibits proteins that bind to PCNA that are necessary for DNA replication and repair, resulting in eventual cellular death. The R9-cc-caPeptide was cytotoxic to several breast cancer cell lines as well as pancreatic cancer and lymphoma. Because the R9-cc-caPeptide is specific to cancer-associated PCNA, it is targeted to cells containing only this isoform and thus has less of an effect on normal cells (Smith et al., 2015).

## A small molecule as an inhibitor of PCNA

T2AA is a small molecule analog of triiodothyronine (T3) that inhibits translesion DNA synthesis (Punchihewa et al., 2012). T2AA was found to inhibit PCNA interaction with p21 and Polδ by binding in the PIP site (Figure 3B). From the crystal structure, it was found that two T2AA molecules bind to each monomer of PCNA with the second molecule binding at the interface between the trimer subunits near K_164_ (Inoue et al., 2014). This lysine residue is a known site for mono-ubiquitination, which is a key factor in regulating how cells respond to DNA damage (discussed in more detail in Choe and Moldovan, 2016). It was reported that T2AA inhibits protein-protein interactions between mono-ubiquitinated PCNA and a polη fragment containing a PIP-box. Inter-strand DNA cross-links are repaired by TLS and monoubiquitinated PCNA. Cells that were treated with T2AA as well as with the cancer therapeutic cisplatin, showed lower survival and an increase in double-strand breaks due to cisplatin. Thus, it is possible that mono-ubiquitinated PCNA could be a drug target for chemo-sensitization with cancer therapeutics (Inoue et al., 2014).

## A DNA aptamer that inhibits DNA polymerase δ and ε

Another direction for the development of anti-cancer drugs is to make use of nucleic acid aptamers. A short DNA aptamer, called α-PCNA, was designed specifically to bind human PCNA that inhibited Polδ and Polε at nanomolar concentrations *in vitro* (Kowalska et al., 2018). The α-PCNA aptamer adopts a β-form helical DNA conformation by itself, but shows some secondary structural changes when bound to PCNA. The PCNA protein itself does not change conformation when α-PCNA binds. It was proposed that an α-PCNA aptamer-PCNA-DNA polymerase complex is not accessible to the primer-template junction on the lagging strand (Kowalska et al., 2018). If the hypothesis is correct than this is a unique mechanism for inhibition of PCNA dependent functions and has potential for future anti-cancer therapy.

## Targeting PCNA in anti-inflammatory treatment

A less explored function of PCNA is its function in inflammatory diseases *via* the role it plays in neutrophil survival. Mature neutrophils are non-proliferating cells and PCNA is found in the cytosol. The mechanism involved is not well-understood, but it has been shown that PCNA binds to several procaspases, preventing their activation and inhibiting apoptosis (Dibbert et al., 1999; Witko-Sarsat et al., 2010). One of the characteristics of cystic fibrosis is an intense pulmonary inflammation that involves neutrophils (Chiara et al., 2012). Neutrophils from patients with this disease experience delayed apoptosis. As an infectious disorder, treatment of cystic fibrosis lung disease involves antibiotics and mucolytics, but this treatment is often marginally successful (Pier, 2012). Anti-inflammatory drugs appear to somewhat delay disease progression. The C-terminal p21 peptide which contains a PIP motif and binds at the PIP site on PCNA, also causes neutrophil apoptosis and subsequent PCNA breakdown (Witko-Sarsat et al., 2010). It has been suggested that targeting PCNA to modulate delayed neutrophil apoptosis, in combination with anti-inflammatory and anti-infectious therapies could be beneficial, but requires more development and research (Chiara et al., 2012).

## Considerations for therapeutics targeting PCNA

Human PCNA is a very stable structure that does not change much when PIP or APIM ligands bind, as the structures are nearly super-imposable with or without ligands. The r.m.s.d. between Cα atoms is < 1 Å for the bound and non-bound structures (Bruning and Shamoo, 2004). Because the IDCL interacts with the PIP peptides, its structure varies with different peptides in the pocket. Most of the PCNA inhibitors reported to bind at the PIP-site do not affect the structure of PCNA. A small protein that can break the trimeric ring of PCNA was reported in the archaeon *Thermococcus kodakarensis* (Li et al., 2014). The protein, referred to as TIP, contains a non-canonical PIP motif that is followed by a 17-residue amphipathic helix that binds on the surface of PCNA near the IDCL. The structural effect of TIP binding to the individual PCNA domains is small, but enough to break apart the PCNA trimeric structure (Altieri et al., 2016). It is not known if a similar protein is expressed in eukarya. PCNA is expressed in all cells and therefore PCNA inhibitors can be toxic not only to the malignant cells but also to the healthy cells. Targeting cytosolic PCNA or specific PCNA variants are likely to be more successful as therapeutics. In their review, de March and de Biaisio (De March and De Biaisio, 2017) discuss structural details of their work on the inner sliding surface of PCNA and suggest targeting these interactions has the potential for new therapeutics. Targeting PCNA in its role in DNA repair and post-translational signaling is likely to enhance specificity to cells involved in disease states (Wang, 2014). PCNA is post-translationally modified by ubiquitination, sumoylation, acetylation, and phosphorylation among others and these modifications can be used as drug targets. PCNA responds to DNA damage by providing an error-free pathway and so adding inhibitors designed to target modified PCNAs could be used in conjunction with anti-cancer therapeutics (Zhu et al., 2014).

## Concluding remarks

The DNA sliding clamps are essential for cell viability and thus are targets for anticancer and antibacterial drugs. Although several peptides and small molecules that inhibit the sliding clamps have been reported, to date none have reached the clinic. One of the main issues with PCNA inhibitors is potential toxicity to healthy cells due to lack of specificity to malignant cells. Future studies may identify new mechanisms to direct drugs only to malignant cells (i.e., antibody-drug conjugates or to post-translational modifications on PCNA in DNA repair). Work on the inhibition of bacterial β-clamps has provided more detailed information and is very promising for the development of new anti-bacterial drugs. Although the overall structures of PCNA and the β-clamps are similar, their amino acid sequences and binding sites are substantially different. Thus, inhibitors that bind the bacterial clamps are not likely to affect eukaryotic PCNA function. Though the research summarized here has moved us forward in DNA clamp inhibition and the design of new anti-bacterial and anti-cancer therapeutics, future work toward a better understanding of the specific mechanisms behind these interactions and related processes will provide much needed insight. It is clear that fundamental research into the structure and binding of therapeutic drugs to PCNA and β-clamp has led to promising advancements in the areas of infectious disease and cancer. Further work towards a better understanding of the specific mechanisms behind the interactions and related processes of sliding clamps promises to provide important applications.

## Author contributions

AA reviewed the literature, summarized the findings, and wrote the paper. ZK suggested the review topic and wrote the paper.

### Conflict of interest statement

The authors declare that the research was conducted in the absence of any commercial or financial relationships that could be construed as a potential conflict of interest.

## Abbreviations

PCNA: proliferating cell nuclear antigen
RFC: replication factor C
FEN-1: flap endonuclease 1
Polδ: DNA polymerase δ
Polε: DNA polymerase ε
Pol III: bacterial DNA polymerase III
PIP: PCNA interacting peptide
APIM: AlkB homolog 2 PCNA-interacting motif
CBM: β-clamp binding motif
IDCL: interdomain connector loop
Cha: cyclohexyl-alanine
GM, Griselimycin**;** CGM: Pro-8-cyclohexyl Griselimycin
NSAIDs: non-steroidal anti-inflammatory drugs
MIC: minimal inhibitory concentration
caPCNA: cancer-associated PCNA
α-PCNA: DNA aptamer of PCNA
TIP: Thermococcales inhibitor of PCNA.
